# Supplementary material for: Efficient Removal of Siloxane from Biogas by Using β-Cyclodextrin-Modified Reduced Graphene Oxide Aerogels
Source: Nanomaterials (Basel). 2022 Jul 31;12(15):2643. doi: 10.3390/nano12152643 (PMC9370590; doi:10.3390/nano12152643)
Supplement: Supplementary file 1 [file nanomaterials-12-02643-s001.zip › nanomaterials-1826258-supplementary.pdf]

Supporting Information

Efficient Removal of Siloxane from Biogas by Using *β*-Cyclodextrin-Modified Reduced Graphene Oxide Aerogels

Yanhui Zheng [1,2], Xifeng Hou [1], Siqi Lv [1], Zichuan Ma [1,*] and Xiaolong Ma [3,*]

1 Hebei Key Laboratory of Inorganic Nano-Materilas, College of Chemistry and Material Science, Hebei Normal University, Shijiazhuang 050024, China; zhengyh0308@163.com (Y.Z.); xifenghoucc@163.com (X.H.); l15231059127@163.com (S.L.)

2 Department of Preshool and Art Education, Shijiazhuang Vocational College of Finance & Economics, Shijiazhuang 050061, China

3 School of Environmental Science and Engineering, Hebei University of Science and Technology, Shijiazhuang 050018, China

* Correspondence: mazc@hebtu.edu.cn (Z.M.); maxiaolong2410@hebust.edu.cn (X.M.); Tel.: +86-311-80787400 (Z.M.)

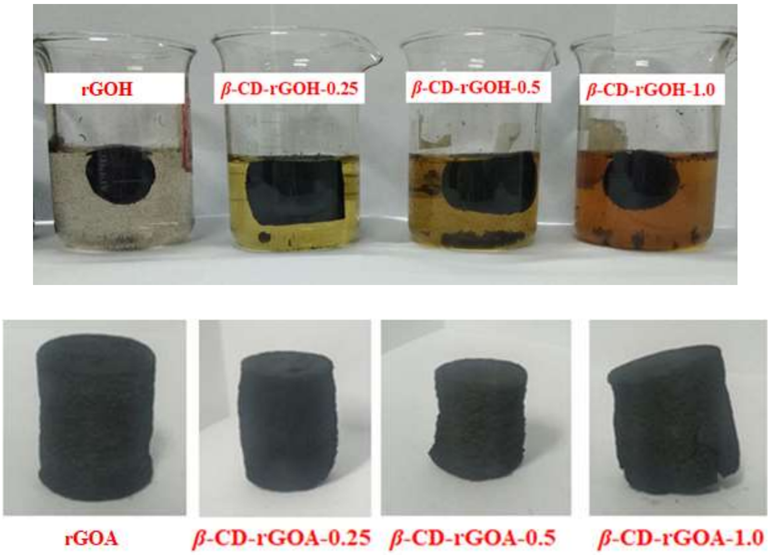

Figure S1. Photographs of of the rGOHs and rGOAs.

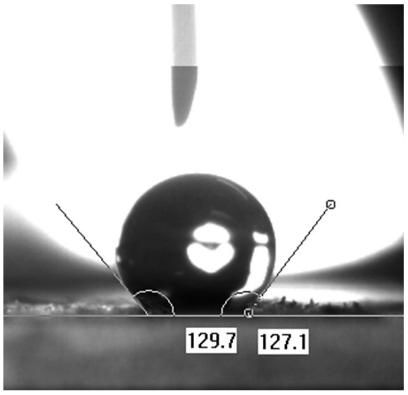

Figure S2. Image showing a water droplet on the surface of *β*-CD-rGOA-0.5 film.

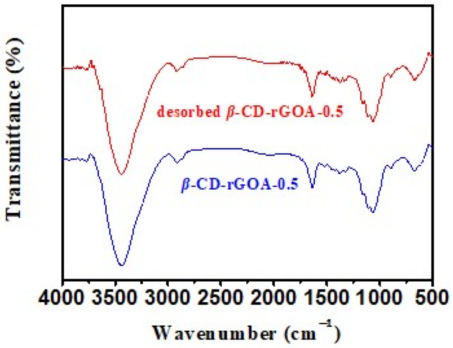

Figure S3. FTIR spectra of *β*-CD-rGOA-0.5 and desorbed *β*-CD-rGOA-0.5 after cycling.

Table S1. Properties of aerogels with different sizes.

samples	Weight (g)	Diameter (cm)	Height(cm)	Volume(cm^3)	Density (mg cm^{-3})
rGOA	0.5681	3.52	4.57	44.44994	12.78
β-CD-rGOA-0.25	0.4032	3.19	4.18	33.39084	12.08
β-CD-rGOA-0. 5	0.4012	3.23	4.21	34.47917	11.64
β-CD-rGOA-1	0.4019	3.50	4.14	39.81128	10.10

Table S2. The statistical parameters of curves between Q_B and S_{BET}, Q_B and V_{tot}, Q_B and θ for the rGOAs.

Statistical parameters		Q_B and S_{BET}	Q_B and V_{tot}	Q_B and θ
Number of Points		5	5	5
Equation			$y = a + bx$	
Residual Sum of Squares		34.9524	337.5752	1118.2794
standard deviation		2.9560	9.1866	16.7203
R-Square		0.9872	0.8760	0.5893
Intercept	Value	6.5455	-23.4917	-84.7256
	Standard Error	2.8039	14.0177	51.8007
Slope	Value	0.5067	151.1688	1.1511
	Standard Error	0.0288	27.9462	0.4435
